# Psychometric properties of the Sinhala perceived stress questionnaire (PSQ8-11) in Sri Lankan primary school children

**DOI:** 10.3389/fpsyg.2024.1357974

**Published:** 2024-09-20

**Authors:** D. L. I. H. K. Peiris, Yanping Duan, Corneel Vandelanotte, Wei Liang

**Affiliations:** ^1^Department of Sport, Physical Education and Health, Faculty of Social Sciences, Hong Kong Baptist University, Hong Kong, China; ^2^Physical Activity Research Group, School of Health, Medical and Applied Sciences, Central Queensland University, Rockhampton, QLD, Australia; ^3^School of Physical Education, Shenzhen University, Shenzhen, Guangdong, China

**Keywords:** childhood, gender, mental health, perceived stress, preadolescents, primary education, validity

## Abstract

**Background:**

Stress influences examination performance among Sri Lankan students. Validated tests are required to evaluate stress levels among elementary students in Sri Lanka. Therefore, the Perceived Stress Questionnaire 8–11 (PSQ8-11) was translated into a Sinhala version. The aim of this study was to examine the psychometric properties of the translated and adapted scale among elementary level school children in Sri Lanka and examine invariance across male and female children.

**Methods:**

The participants were 1021 students from seven schools. After removing missing values, responses from 693 students (mean age = 9.65 ± 0.478 years, 51.8% male) were analysed for participant characteristics. Cronbach’s alpha, Spearman’s correlation, and confirmatory factor analysis with measurement invariance models were conducted after adding one item to the original PSQ8-11 version.

**Results:**

The Cronbach’s alpha value for the 20-item modified PSQ8-11 Sinhala version was.788. The two subscales, psychological stress (Cronbach’s alpha = 0.615) and physiological stress (Cronbach’s alpha = 0.711), indicated a satisfactory level of internal consistency. Furthermore, a statistically significant correlation (*p* < 0.01; 2-tailed) was reported among each of the subscales. Confirmatory factor analyses demonstrated a satisfactory goodness-of-fit across the two models by confirming the theoretical constructs of the PSQ8-11 translated version with its two subscales. The two-factor model has better model fit indices compared to the unidimensional model (χ^2^/df = 1.447, CFI = 0.947, TLI = 0.938, WRMR = 0.028, RMSEA = 0.026, SRMSR = 0.0341, and PCLOSE = 1 of the two-factor model). Measurement variance across gender was supported by the establishment of configural and metric invariances.

**Conclusion:**

Acceptable psychometric properties for the PSQ8-11 Sinhala version were observed in elementary schoolers in Sri Lanka.

## 1 Introduction

Stress is a critical factor affecting the psychological and physiological wellbeing, as well as the academic achievement, of preadolescents (Snoeren and Hoefnagels, 2014; Whiting et al., 2021). Stress among preadolescent students in Sri Lanka is a critical issue (Sedere, 2010; Wasantha, 2015; Hamilton et al., 2016; Sedere et al., 2016; Peiris et al., 2023) that needs to be assessed through robust measures (Hamilton et al., 2016). Self-reported measures to assess perceived stress are preferable (Snoeren and Hoefnagels, 2014; Linden and Stuart, 2019; Manzar et al., 2019; Useche et al., 2021; Wong et al., 2022), as measuring stress levels through neurobiological methods is challenging and resource consuming (Useche et al., 2021; Wong et al., 2022). However, more research is needed to collect self-reported responses directly from children to assess their stress levels, as using proxies (e.g., parents) reveals many inconsistencies (Davis et al., 2007; Koning et al., 2018; Germain et al., 2019). Piaget’s stages of development theory confirm the literacy ability of a seven-year-old (Piaget, 1977; Winstanley, 2022) and thus the ability to directly assess perceived stress in children over the age of seven.

A systematic review on perceived stress among children between 8 and 12 years old (Davis and Soistmann, 2022) showed that the majority of included studies relied on parents or clinicians or that they measured concepts related to perceived stress (e.g., physical responses to stress and stressful life events) but not actual perceived stress. Furthermore, some self-report measures in these studies were only validated in children older than 12 but were not validated in younger children. Only four questionnaires (Feel Bad Scale with 40 items, Hassles Scale for Children with 43 items, Stress Events Perception Scale with 24 items and Perceived Stress Questionnaire 8–11 with 19 items) were identified by the systematic review (Davis and Soistmann, 2022) as validated self-reported instruments exclusively for use among children younger than 12 years old. Of these measures, only the Perceived Stress Questionnaire 8–11 (PSQ8-11) had a subscale to measure both the physiological and psychological aspects of stress (Snoeren and Hoefnagels, 2014). Furthermore, compared to the other three self-reporting tools, the PSQ8-11 had the least number of items in the questionnaire, which is more ideal for a younger age group, who can be easily distracted away from concentrating on answering lengthy lists of questions (Davis and Soistmann, 2022). The systematic review (Davis and Soistmann, 2022) also highlighted the need for establishing the psychometric properties of the PSQ8-11 in other populations due to its existing validation and the feasibility of utilisation among preadolescents.

The PSQ8-11 is a well-established scale (Snoeren and Hoefnagels, 2014; Razani et al., 2019; Davis and Soistmann, 2022; Davis and Turner-Cobb, 2023) to assess self-reported stress levels, including both psychological and physiological stress, in eight- to eleven-year-old children. The PSQ8-11 was developed in the Netherlands (for Dutch students) by modifying an existing questionnaire (i.e., the Maastricht University Stress Instrument for Children above 12 years old) and consists of two sub scales focusing on psychological stress and physiological stress (Snoeren and Hoefnagels, 2014). Each subscale consists of nine and ten items, respectively. Its psychometric properties were established in a study with 123 boys and 100 girls (mean age = 9.5 years, SD ± 0.9) from three primary schools (Snoeren and Hoefnagels, 2014). The internal consistency of the PSQ8-11 was high for the complete scale (Cronbatch’s α = 0.85), as well as for the subscales of psychological stress (Cronbatch’s α = 0.76) and physiological stress (Cronbatch’s α = 0.80). The psychometric properties of PSQ8-11 (through internal consistency, test-retest reliability and exploratory factor analysis) have been preliminarily verified in Turkey (Oral and Ersan, 2017) with an adapted version according to Kacar and Alkaya (Kacar and Ayaz-Alkaya, 2022). However, there are some limitations in the adapted version such as its structural validity and the measurement invariance. Researchers (Razani et al., 2019) reported that the validity measures have not yet been completed PSQ8-11. Therefore, further validation processes should be followed up on the PSQ8-11 among different cultures and countries such as Sri Lanka.

According to our knowledge, there are no validated assessment tools available to examine the perceived stress levels in Sri Lankan students under 11 years of age even though these children are assumed to be stressed over academic burden (Amarasinghe, 2014; Abayasekara, 2019; Peiris et al., 2023, 2024). It is critically discussed that the Sri Lankan primary school students, specially the fifth graders are stressed as they are required to face a national level grade five scholarship examination in addition to their curriculum-related term tests. However, such claims are based on the qualitative findings based on proxy statements such as educators and parents. Therefore, it is important to obtain a validated self-assessment from the students to validate the claims of their stress levels.

We translated the PSQ8-11 (Snoeren and Hoefnagels, 2014) into a Sinhala version. The aim of this study was to examine the psychometric properties of the Sinhala version of the PSQ8-11, including its internal consistency, test-retest reliability, and construct validity. In addition, the study also aimed to examine the measurement invariance across male and female children. Because the case where a psychological instrument assesses an unobserved construct in the same manner for different groups of individuals or across time was not evident in the original study. Thus, the translated PSQ8-11 scale will exhibit measurement invariance with respect to gender if its items measure perceived stress identically for males and females. Likewise, if such a measure to the same individuals at two points in time were given, it will be equally reflective of perceived stress in both instances. However, it is critical to note that measurement invariance does not imply the same level of performance at both times or for males and females (Finch, 2014). Rather, it simply means that the PSQ8-11 scale is equally reflective of the construct of interest in all instances.

Furthermore, a systematic review (Davis and Soistmann, 2022) implied that the differences between male and female elementary level students’ perceived stress was not evident, few studies (Gokkaya and Kargul, 2020; Radwan et al., 2021) revealed that the perceived stress of girls can be higher than boys among 11 to 18-year-old students. Thus, the findings on measurement invariance would be a great contribution to the future studies.

## 2 Methods

This cross-sectional study was conducted with research ethics approval from the Ethics Review Committee of the University of Kelaniya, Sri Lanka (Ref: UOK/ERC/SS/2022/009) and the Hong Kong Baptist University’s Research Ethics Committee (Ref: SOSC-SPEH-2022-23_113).

### 2.1 Translation and adaptation of the PSQ8-11 into Sinhala

A standard scale translation protocol (Hambleton et al., 2004) was applied to translate the PSQ8-11 questionnaire into Sinhala. Two independent translations of the PSQ8-11 questionnaire (English version) into Sinhala were made. After the initial translations were completed, both versions were compared and discussed by the translators until consensus was reached on any discrepancies. Then, a back-translation was conducted by two other bilingual experts using a standard back-translation technique (McKay et al., 1996). After the translation and back-translation work, the translated PSQ8-11 was provided to an expert panel of three fifth grade teachers for comments and discussion. The three fifth-grade teachers were chosen as experts because they had more than twenty years of trained teaching experience with primary school students in Sri Lanka. Two of the female teachers were from Uva Province, and the other male teacher was from the Northwestern province. Based on these teachers’ work experiences, fluency in English and Sinhala languages, and their current engagement with elementary graders as class teachers, they were well positioned to identify any discrepancies in the words of choice of the translated questionnaire. All three teachers had experience working with all five levels (grades 1 to 5) of the primary schools as class teachers during their tenure. Upon receiving the feedback, several modifications were made, including changing the spelling of three words, increasing the font size of the questionnaire, using emojis and applying a different colour to the important words of each question.

It was reported that the fifth graders in Sri Lankan primary schools are experiencing higher perceived stress levels due the national level fifth-grade scholarship examination that the children should face (Peiris et al., 2023, 2024). Compared to grade one to four, fifth grade teachers use longer seated learning hours, and practice examinations, which may lead to the distress of the students (Peiris et al., 2023, 2024). Therefore, 21 fifth-grade teachers were interviewed. The underlying reason behind the interview was to identify stress-related observations that were not assessed in the original version of the PSQ8-11. The interview guide and interviewees’ characteristics are published elsewhere (Peiris et al., 2023). In summary, the interview guide was consisted with semi-structured questions such as “Do you think that the students are suffering from mental-health problems such as stress because of the Scholarship examination? Can you tell me why do you think so? May you share some experiences on how you got to know that they are experiencing stress?… A content analysis was performed using QDA Miner Lite software by Provalis Research, Canada. A low level of interest in attending school was identified as another indicator of psychological stress among elementary school children in Sri Lanka. For example, a 45-year-old female teacher said:

“…*because there is too much bookwork. They do after-school classes continuously in the evening too, and then that child cannot bear it. Because of the pressure, the child cannot stay focused in the class and becomes stressed. Consequently, children refuse to go to school because of that?”*

As a result, one extra item was added to the psychological subscale of the translated Sinhala PSQ8-11 questionnaire (i.e., “In the last week, how often did you feel like not going to school?”). The same expert panel also provided their recommendation towards the added item. Finally, the 20-item Sinhala PSQ8-11 questionnaire was read through by four fifth-grade students (two girls and two boys) in government primary school in Sri Lanka to test its understandability. All students reported that each item of the questionnaire was readable and understandable.

### 2.2 Participants and sampling

**FIGURE 1:**
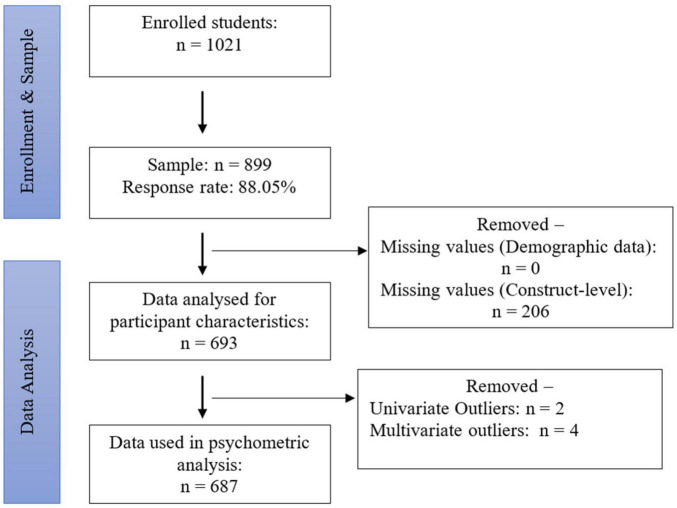
Flow diagram of the study sample.

Fourth- and fifth-grade students (ages 9 to 10 years old) attending government primary schools in Sri Lanka were targeted in this study. According to the sample size rule of thumb for confirmatory factor analysis (Wolf et al., 2013), the minimum sample size is suggested to be 10 times the number of items included in the questionnaire. As the PSQ8-11 had 19 items in the original version (original questionnaire items can be found in Supplementary Material 1) and one additional item was introduced to the translated version (the Sinhala version of PSQ8-11 can be found in Supplementary Material 2), the minimum required sample was 200 students. Based on the data shared by the Ministry of Health, Sri Lanka, on the spread and severity of the COVID-19 pandemic, Badulla District was less affected by the pandemic. Therefore, the students were recruited from Badulla District based on the conveniency to travel and the severity of the pandemic spread from June to July 2022. Because, in addition to the pandemic, the economic crisis in Sri Lanka caused halt or reduced transportation means due to gasoline deficits. Verbal consent to collect data from 1021 students from seven schools was received (Figure 1). Due to the economic-crisis in Sri Lanka, the principal investigator faced an issue of finding enough papers to print all the consent documents. Therefore, we prioritized our needs to save the limited papers as it was difficult to find papers to print the questionnaires.

After removing missing data, responses from 693 students were analysed for participant characteristics. There were 359 male students (51.8%) and 334 female students in the sample; 68% of the sample (n = 471) were from fifth grade, 64.9% (n = 450) were 10 years old, and 37.7% were nine years old. The mean age of the participants was 9.65 (SD = 0.478). As two serious univariate (> ±3.29) and four multivariate outliers (Mahalanobis distance: χ2 = 22.46, df (6), *P* < 0.001) were detected, only 687 were included in the psychometric analysis.

### 2.3 Inclusion and exclusion criteria

To be included in the study, the students had to attend a government primary school in Sri Lanka, where the language of instruction is Sinhala. Their age ranged from 8 to 11 years old, as the PSQ8-11 was developed for that specific age group (Snoeren and Hoefnagels, 2014). Students with an inability to read or write, as well as those with any special need or psychological disorders, were excluded from the sample after consulting with the teachers.

### 2.4 Measures

The original version of the PSQ8-11 (Snoeren and Hoefnagels, 2014) consists of 19 items and includes two subscales: psychological stress (9 items) and physiological stress (10 items). As one item was added to the psychological stress-related measures for the Sinhala PSQ8-11 questionnaire (i.e., “In the last week, how often did you feel like not going to school?”), the Sinhala version of the PSQ8-11 consisted of 20 items. Each subscale requires the students to recall their feelings from the previous week.

The psychological stress-related 9 items of PSQ8-11 consisted of questions such as “In the last week, how often did you find it hard to calm down?” and “In the last week, how often did you easily become sad?”. The physiological stress-related 10 items consisted of questions such as “In the last week, how often did you have a stomach-ache?” and “In the last week, how often did you feel dizzy?” The answers were given on a 4-point Likert scale (1 = never, 2 = sometimes, 3 = often, 4 = very often). A higher score on the questionnaire corresponds with greater perceived stress.

### 2.5 Procedure(s)

The questionnaires were directly distributed to classroom teachers and their students. The principal investigator or a research assistant explained the consent forms to the students and teachers before the students started completing the questionnaire. Then, the students, as well as the teachers (as the guardians of each student), provided verbal consent to complete the questionnaire. Completing the questionnaire took approximately 15 minutes (including the time to explain the information of the consent form), as teachers guided the students by reading questions out loud in the classrooms. However, the teachers did not interfere with the student responses, and they ensured that they provided responses voluntarily. A retest was conducted by the students after a one-week interval.

### 2.6 Data analysis

IBM SPSS software Statistics Version 27 was used to analyse the data. Before conducting the descriptive analysis and model evaluation, diagnostic tests such as exploring data distribution and missing value patterns were carried out.

The internal consistency and composite reliability of the questionnaire were evaluated through Cronbach’s alpha statistics and test-retest reliability. Based on a recent recommendation (Taber, 2018) with regard to alpha value interpretations, a moderate (Cronbach’s alpha = 0.61–0.65) to fairly high (Cronbach’s alpha = 0.76–0.95) alpha value was expected to imply the adequacy of the items’ internal consistency. Test-retest reliability was examined through the Pearson correlation test, [expecting a moderate (± 0.50 to.70) to very high (± 0.90 to 1.00) correlation (Mukaka, 2012)] and the intraclass correlations (ICC) expecting a good [0.6 to 7.4 to excellent (> 0.75) scale (Ćwirlej-Sozańska et al., 2020)]. Pearson correlation was preferred over the reporting of intraclass correlations (ICC), as our intention was to calculate the performance from test to retest instead of calculating both consistency of performances from test to retest, as well as change in average performance of participants as a group over time (Vaz et al., 2013). Additionally, the ICC is used frequently to calculate the correlation between more than two sets of measurements (Vaz et al., 2013). The current study did not have more than two sets of measures. Therefore, Pearson correlation was used for the convenience of accurately comparing the correlation levels of the original study with the current study. However, the ICC was also reported for further validation.

Confirmatory factor analyses (CFAs) were employed to examine the construct validity of the PSQ8-11 translated version using IBM SPSS AMOS version 27. The goodness-of-fit of each model generated through CFA was confirmed when the chi-square to degree of freedom ratio (χ^2^/df) value ranged from 2 to 5 (Kline, 2016), when the Comparative-Fit Index (CFI) was less than 1, when the Tucker-Lewis Index (TLI) was greater than 0.90, when the Weighted Root Mean Square Residual (WRMSR) was smaller than 0.5, when the Root Mean Square Error of Approximation (RMSEA) was smaller than 0.08, when the Standardised Root Means Square Residual (SRMSR) was smaller than 0.08 and the Probability of Close Fit (PCLOSE) was greater than.05 (Kuan et al., 2019; Linden and Stuart, 2019; Manzar et al., 2019; Ćwirlej-Sozańska et al., 2020; Useche et al., 2021).

The original version of the PSQ8-11 did not examine measurement invariance across the gender of the students. Thus, Mplus 8 software was used to examine the measurement invariance across gender. Multiple-group CFAs were performed to identify the measurement invariance across gender. Four distinctive levels of measurement invariance were examined by progressively constraining the parameter estimates of the models to be equivalent across the samples: (1) configural invariance, where no parameter estimates were restricted to equality; (2) metric invariance, where factor loadings were constrained to equality; (3) strong invariance, where both factor loadings and item intercepts were constrained to equality; and (4) structural/strict invariance, where all factor loadings, item intercepts, and factor variance and covariance were restricted to equality (Liang et al., 2020). The measurement invariance would be supported if the changes in the value of CFI and RMSEA were at ≤0.01 and ≤0.015, respectively (Liang et al., 2020). The change in the value of CFI and RMSEA between different nested samples at *p* ≤ 0.01 and *p* ≤ 0.015 would respectively indicate support for invariance (Conijn et al., 2020; Liang et al., 2020).

## 3 Results

The diagnostic tests indicated that several PSQ8-11 items departed from the normal distribution, and the absolute values of skewness and kurtosis were > 1. Either a ceiling or floor effect was not detected, as the percentage of the extreme values did not exceed more than 15% of the respondents in the sample (Manzar et al., 2019). Therefore, it was confirmed that the robust maximum likelihood estimation could be performed in the confirmatory factor analysis, whereby the standard errors and tests of model fit can be robust with respect to the observed variables with non-normal and non-independent distributions (Liang et al., 2020). The missing value analysis indicated that construct-level data were missing in a random pattern from 206 respondents (Figure 1), which accumulated to more than 5% of the total data. Thus, those missing data were excluded from the analysis of participant characteristics (Manzar et al., 2019) and analysed 687 data for psychometric properties.

### 3.1 Internal consistency and test-retest reliability

The Cronbach’s alpha value of the 20-item PSQ8-11 Sinhala version was.788. The two subscales for psychological stress (Cronbach’s alpha = 0.615) and physiological stress (Cronbach’s alpha = 0.711) also indicated a satisfactory level of internal consistency. The composite reliability was 0.787. Furthermore, Table 1 indicates a sufficient level of statistically significant correlation among each of the subscales, and the correlation was significant at the 0.01 level (2-tailed). For the overall result, ICC for the PSQ8-11 was 0.777, which confirmed that the scale was consistent over time.

**TABLE 1:** Correlations Matrix for PSSQ8-11 and Subscales.

	Test1_ Psychological	Test1_ Physiological	Test1_ Overall	Test2_ Psychological	Test2_ Physiological	Test2_ Overall
Test1_Psychological	1					
Test1_Physiological	0.583^**^	1				
Test1_Overall	0.881^**^	0.898^**^	1			
Test2_ Psychological	0.651^**^	0.597^**^	0.700^**^	1		
Test2_ Physiological	0.481^**^	0.676^**^	0.655^**^	0.694^**^	1	
Test2_Overall	0.612^**^	693^**^	0.735^**^	0.915^**^	0.925^**^	1
ICC for average measures 0.927^**^						

**Correlation is significant at the 0.01 level (2-tailed).

### 3.2 Construct validity

**FIGURE 2:**
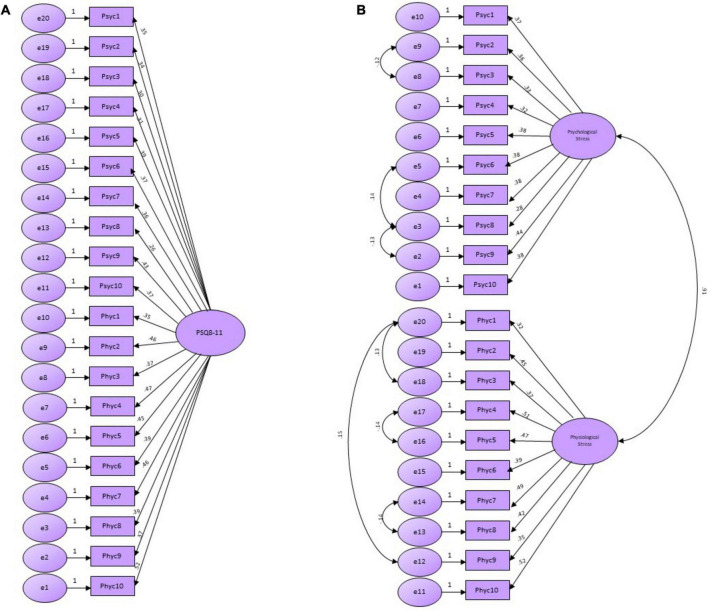
**(A)** Confirmatory factor analysis models of the PSQ8-11. **(B)** Confirmatory factor analysis models of the PSQ8-11.

Construct validity through the CFA was examined using a unidimensional model (Model A) and a two-factor model (Model B) across the study sample, as shown in Figures 2A, B. The model fit indices demonstrated (Table 2) a satisfactory goodness-of-fit across the two models by confirming the theoretical constructs of the PSQ8-11 Sinhala version with its two subscales. Furthermore, the results (Table 2) indicated that the two-factor model has better model fit indices than the unidimensional model.

**TABLE 2:** Model fit statistics of the translated PSQ8-11 among fifth graders.

PSQ8-11	CFI	TLI	RMSEA (90% CI)	WRMSR	SRMSR	PCLOSE	X^2^	df	*P*	X^2^/df
Model A	0.891	0.878	0.036 (0.030,0.042)	0.033	0.0397	1.000	318.640	170	< 0.001	1.874
Model B	0.947	0.938	0.026 (0.018,0.032)	0.028	0.0341	1.000	234.465	162	< 0.001	1.447

Model A: Unidimensional model, Model B: Two-factor model.

### 3.3 Measurement invariant model

The measurement invariance of the translated PSQ8-11 was examined based on the gender of the students. Based on the configural invariant test (Table 3) for the unconstrained model, an adequate goodness-of-fit was identified when analysing freely across two groups: male and female. i.e., The change in the value of CFI between the configural, metric and strict samples was ≤ 0.01. The change in the value of RMSEA between all models were ≤ 0.015. Thus, the multiple-group CFAs demonstrated that the 20-item two-dimensional Sinhala PSQ8-11 scale to be invariant at both configural and metric levels for all samples.

**TABLE 3:** Summary of goodness of fit of the measurement invariance analysis models.

Model	Chi-square (*df*)	*P*	CFI	Δ CFI^a^	TLI	Δ TLI^b^	RMSEA (90% CI)	Δ RMSEA^c^
Configural	425.536 (332)	<0.001	0.915	N/A^d^	0.902	N/A	0.029 (0.020, 0.036)	N/A
Metric	450.940 (350)	<0.001	0.908	0.007	0.900	0.002	0.029 (0.020, 0.036)	0
Strong	510.889 (370)	<0.001	0.872	0.036	0.868	0.032	0.033 (0.026, 0.040)	0.004
Strict	516.280 (373)	<0.001	0.869	0.003	0.867	0.001	0.033 (0.026, 0.040)	0.004

^a^Δ CFI, change in the CFI; ^b^Δ TLI, change in the TLI; ^c^Δ RMSEA, change in the RMSEA; ^d^N/A, not applicable.

## 4 Discussion

This study is the first to translate and examine the psychometric properties of the self-reported PSQ8-11 among elementary level school children in Sri Lanka. The study was able to recruit more than three times (n = 687) the minimum sample requirement (n = 200). The Cronbach’s alpha value of the 20-item PSQ8-11 Sinhala version indicated a satisfactory level of internal consistency. A sufficient level of statistically significant correlation among each of the subscales demonstrated the test-retest reliability of the translated version of the PSQ8-11. In terms of the construct validity, the CFA demonstrated a satisfactory goodness-of-fit across a unidimensional model and a two-factor model by confirming the theoretical constructs of the PSQ8-11 translated version with its two subscales. The two-factor model indicated better model fit indices than the unidimensional model. The results indicate that the gender groups are invariant at the model level.

The analyses showed sufficient evidence on the internal consistency reliability. The analyses showed sufficient evidence on the internal consistency reliability (Cronbach’s α = 0.615), which is consistent with the original version of the scale (Cronbach’s α = 0.76). It worth noting that the internal consistency reliability of the PSQ8-11 in our study is lower than the original version. Although many studies (Snoeren and Hoefnagels, 2014; Koning et al., 2018; Conijn et al., 2020) have demonstrated the ability of preadolescents above seven years old to self-report their health-related status, two possible explanations may explain the moderate internal consistency levels for psychological stress. (1) During early ages of preadolescence, children are more likely to refer to physiological experiences to express their stress (Miller et al., 2011; Snoeren and Hoefnagels, 2014; Smith and Pollak, 2020). Piaget’s developmental stages theory (Piaget, 1977; Winstanley, 2022) also confirms that preadolescents’ abstract thought remains difficult even though they develop reading and writing skills by the age of seven. (2) Based on research findings from Japan, (Toyama, 2010, 2011) preadolescents tend to refuse the possibility of having bodily reactions due to psychological distress. Thus, many children believe that physical reactions are solely caused by physiological discomfort rather than psychological distress (Toyama, 2010, 2011; Snoeren and Hoefnagels, 2014). At the same time, recognition of the psychogenic body starts between the ages of eight and eleven (Toyama, 2010, 2011; Snoeren and Hoefnagels, 2014). Therefore, it is possible that the moderate levels of internal consistency for perceived psychological stress among this group are related to confusion in identifying their psychogenic body and its reactions.

The test-retest reliability correlations (r = 0.651_psychological_ to 0.735_physiological_) were also moderate compared to the original survey (*r* = 0.79 _psychological_ to 0.72 _physiological_) conducted in the Netherlands (Snoeren and Hoefnagels, 2014). The literature suggests an inadequate sense of time among this age group (Snoeren and Hoefnagels, 2014; Germain et al., 2019; Conijn et al., 2020). The children may show their ability to recall information about their health but may find it difficult to memories exactly when something related to their health has occurred (Snoeren and Hoefnagels, 2014; Germain et al., 2019; Conijn et al., 2020), resulting in lower test-retest reliability (Allen, 1988; Notaro et al., 2001). Therefore, the results suggest that the participants from the original study may have had a higher ability to recall when the stressors related to their health compared to the participants from Sri Lanka. This may be due to the different social factors that the samples from the two countries were experiencing during the data collection period. For example, the original study did not mention any hindrances that the students faced during their data reporting period. However, the participants from Sri Lanka may have been distracted by the COVID pandemic and economic crisis-related school closures when recalling when psychological stressors occurred. However, it is commending that the students from both the original and Sri Lankan studies reported high levels of correlations for reporting physiological stress by confirming that the targeted age group has the ability to recall information about their health (Toyama, 2010, 2011; Snoeren and Hoefnagels, 2014).

While the original study did not examine construct validity through a CFA, many researchers (Vaughn et al., 2009; Baik et al., 2019; Manzar et al., 2019) have analysed perceived stress questionnaires by comparing univariate models and two-factor models to ensure the construct validity of self-reported tools on stress across different age groups and populations (Manzar et al., 2019; Liang et al., 2020; Wong et al., 2022). The CFA favored the two-factor model (Model B) compared to its univariate model. Thus, this study could confirm the validity of using the two theoretical constructs such as psychological and physiological stress to assess perceived stress among children between 8 and 11 years old. Furthermore, better model fit indices obtained for the two-factor model included the newly added item to the PSQ8-11 Sinhala version. That item was introduced to the subscale on psychological stress based on the frontline teaching staff in Sri Lankan primary schools. Thus, the two-factor model with the added item suggests the suitability of the PSQ8-11 Sinhala version for assessing perceived stress levels among primary school children in South Asian countries, such as Sri Lanka.

Finally, this study examined the measurement invariance of the Sinhala PSQ8-11. Furthermore, the establishment of the metric invariance supporting the equivalence of factor loadings among the samples indicates that the latent factors of the PSQ8-11 were measured in the same way in each of the four samples [43]. Although strong and structural invariance were not supported, the establishment of configural and metric invariances demonstrated that the PSQ-8 is a psychometrically sound instrument for appropriate and meaningful trans-group comparisons between boys and girls. Recent studies have confirmed that perceived stress varies according to gender. For example, findings from a study among 8–11-year-old primary school children (*n* = 171 female students, *n* = 132 male students) in Turkey found that girls tend to report higher total perceived stress than boys (Gokkaya and Kargul, 2020). Another study conducted with school children from 11 to 18 years old (*n* = 385 students) found that girls are more likely to have moderate stress than boys in Palestine (Radwan et al., 2021). Hence, the utilisation of Sinhala PSQ8-11 is needed to examine any differences across gender levels within Sri Lankan students. And more validations on the PSQ 8-11 scale is needed for low- and middle-income countries where there is less evidence about perceived stress among girls and boys.

### 4.1 Strengths and limitations

Preadolescents in Sri Lanka receive school education in three languages: Sinhala, Tamil, and English. The majority of the schools are educated in Sinhala, and this study obtained responses only from the students who received education in Sinhala medium. Therefore, the Sinhala PSQ8-11 will be of no use to students in Tamil and English schools. This study did not focus on differently abled students and students with special needs. Thus, future studies are recommended to address the sample-related limitations of this study.

This study did not assess the convergent/concurrent validity of the translated PSQ8-11 due to the absence of validated self-reported measurements on anxiety or quality of life for preadolescents that can be used as a high-quality comparison. Studies indicated that the Cronbach’s α coefficient and composite reliability scores can be acceptable at 0.7 (Ćwirlej-Sozańska et al., 2020). However, Cronbach’s α of the perceived psychological stress is below 0.7, even though other reliability statistics are above the acceptable score. To proceed with the current study, considering the lower loading scores for the explorative factor analysis from the original PSQ8-11 was another limitation. During the data collection period, there were reduced weekly school days and time allocated for the elementary level kids because of the COVID-pandemic and the economic crisis. Therefore, concurrent validity through any diagnostic clinical interview was not employed. Hence, future studies are proposed to examine the convergent validity of the PSQ8-11.

Despite the limitations mentioned above, the strengths of this study are worth noting. Even though the data were collected during the COVID-19 pandemic, we could recruit a three-times larger sample (*n* = 687) compared to the required minimum number of subjects (*n* = 200) to test the psychometric properties. Moreover, the response rate of the sample was over 88%. Therefore, with a large sample size, it was possible to employ some advanced statistical methods, such as CFA, to test the psychometric properties of the Sinhala version compared to its original version and confirm acceptable levels of psychometric properties for the PSQ8-11 Sinhala version.

In addition to the strengths mentioned above, we had an opportunity to address several limitations mentioned in the original version of the PSQ8-11: (1) this survey was carried out as an additional study to replicate the findings from the original study, (2) we examined the construct validity of the PSQ8-11, (3) we contained a fair representation of both male and female respondents, and (4) we added another child-specific item (for psychological stress) to obtain a more complete view of perceived stress among Sri Lankan children. Additionally, aligning with previous investigations, this study acts as evidence to propose more efforts to prevent proxy errors in data collection by considering children as key informants of their wellbeing-related issues.

## 5 Conclusion

This study provides acceptable evidence for the psychometric properties of the PSQ8-11 among elementary level school children. Therefore, future researchers may utilise the translated Sinhala version of the PSQ8-11 to evaluate perceived stress among 8- to 11-year-old children who can read Sinhala.

## Data availability statement

The raw data supporting the conclusions of this article will be made available by the authors, without undue reservation.

## Ethics statement

The studies involving humans were approved by the Ethics Review Committee of the University of Kelaniya, Sri Lanka (Ref: UOK/ERC/SS/2022/009) Hong Kong Baptist University’s Research Ethics Committee (Ref: SOSC-SPEH-2022-23_113). The studies were conducted in accordance with the local legislation and institutional requirements. Written informed consent for participation in this study was provided by the participants’ legal guardians/next of kin. Written informed consent was obtained from the minor(s)’ legal guardian/next of kin for the publication of any potentially identifiable images or data included in this article.

## Author contributions

DP: Conceptualization, Data curation, Formal analysis, Funding acquisition, Investigation, Methodology, Project administration, Resources, Software, Validation, Visualization, Writing–original draft, Writing–review and editing. YD: Conceptualization, Project administration, Supervision, Validation, Writing – review and editing. CV: Conceptualization, Supervision, Validation, Writing–review and editing. WL: Conceptualization, Methodology, Formal analysis, Supervision, Writing–review and editing.
