# Supplementary material for: Psychometric properties of the Sinhala perceived stress questionnaire (PSQ8-11) in Sri Lankan primary school children
Source: Front Psychol. 2024 Sep 20;15:1357974. doi: 10.3389/fpsyg.2024.1357974 (PMC11451295; doi:10.3389/fpsyg.2024.1357974)
Supplement: Supplementary file 1 [file Table_1.DOCX]

Supplementary Material 1

###
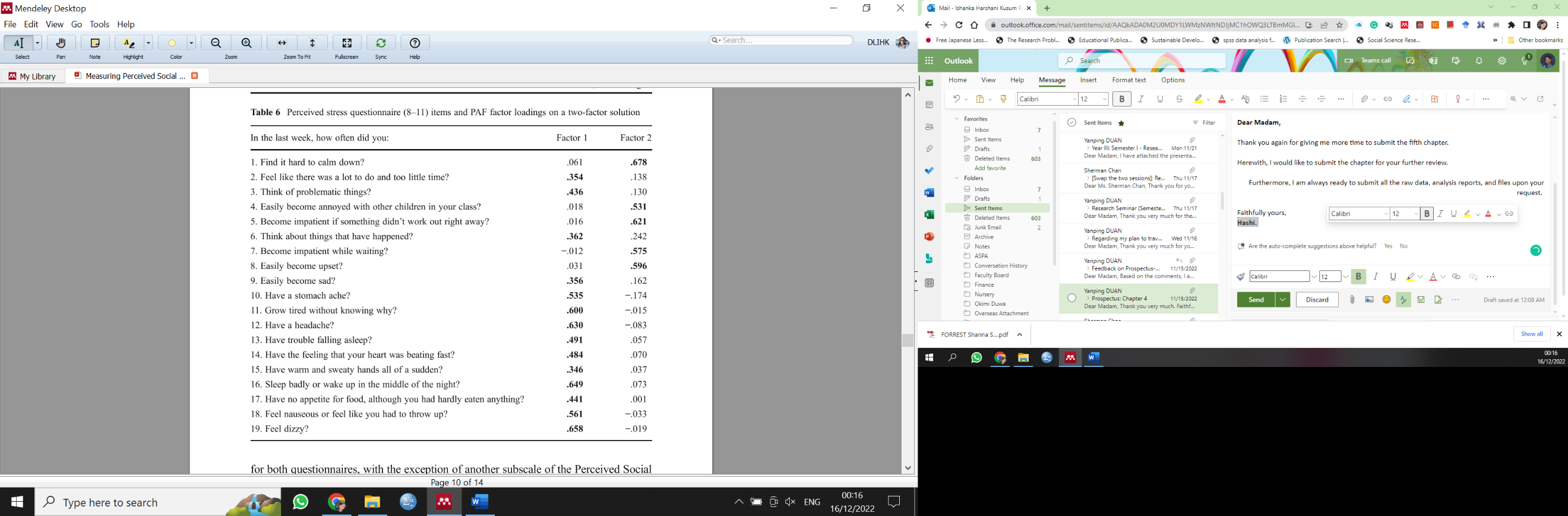
Factor loadings for PSQ8-11 original version

Source: Snoeren & Hoefnagels, 2014

**
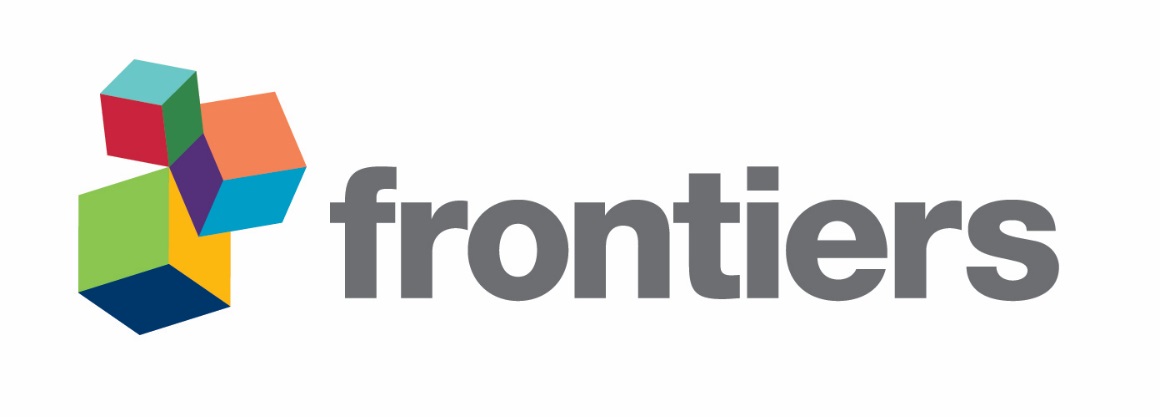
**

### Supplementary Figure 1.
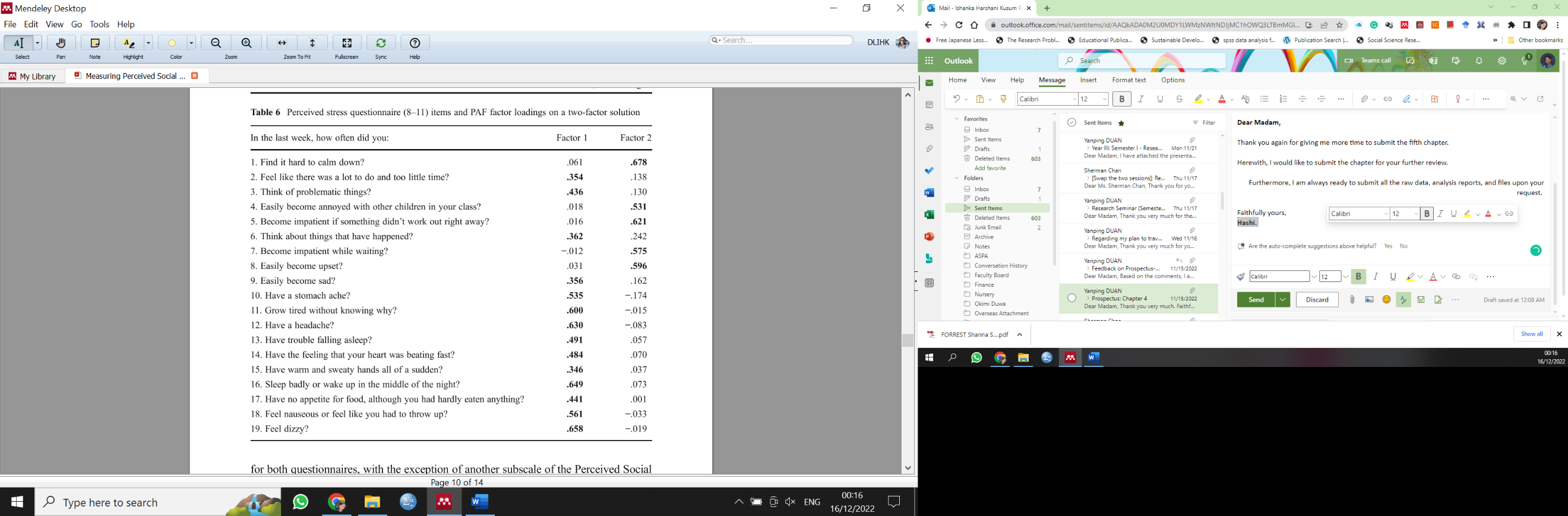
Factor loadings for PSQ8-11 original version

Source: Snoeren & Hoefnagels, 2014

.
