# Supplementary material for: Psychometric properties of the Sinhala perceived stress questionnaire (PSQ8-11) in Sri Lankan primary school children
Source: Front Psychol. 2024 Sep 20;15:1357974. doi: 10.3389/fpsyg.2024.1357974 (PMC11451295; doi:10.3389/fpsyg.2024.1357974)
Supplement: Supplementary file 2 [file Table_2.DOCX]

Supplementary Material 2

දුවේ/පුතේ, පහත වගුවේ සඳහන් ප්‍රකාශ කියවා ඊට ඉදිරියෙන් ඇති කොටුවල ලියා ඇති වචනවලින් ඔබට වඩාත් අදාළ වෙනවා යැයි සිතෙන පිළිතුරු වචනය උඩින් 🗙ක් / කතිර ලකුණ යොදන්න. 😊 (Daughter/Son, read the statements in the table below and put a 🗙 mark above the answer word that you think is most relevant to you from the words written in the boxes in front of it.)

| **ප්‍රකාශය (Statement)** | **වඩාත් ගැළ‌පෙන වචනය උඩින් කතිර ලකුණ යොදන්න. (Place the × above the most suitable word.)** | | | |
| --- | --- | --- | --- | --- |
| 1. **පසුගිය සතියේ**, ඔබට ඔබව සන්සුන්කරවා ගැනීමට කොපමණ වාරයක් අපහසු වූවාද? (In the last week, how often did you find it hard to calm down?) | එහෙම වුණේ නෑ (Never) | සමහර දවස්වලට (Sometimes) | බොහෝ වෙලාවට (Often) | නිතරම වගේ (Very often) |
| 2. **පසුගිය සතියේ**, ඔබට බොහෝ දේ කිරීමට ඇති බවත්, ඒවා කිරීමට ඔබට ඇති කාලය ඉතා අඩු බවත් කොපමණ වාරයක් දැනුණාද? (In the last week, how often did you feel like there was a lot to do and too little time?) | එහෙම වුණේ නෑ (Never) | සමහර දවස්වලට (Sometimes) | බොහෝ වෙලාවට (Often) | නිතරම වගේ (Very often) |
| 3. **පසුගිය සතියේ**, ගැටලු සහගතයැයි දැනෙන දේවල් ගැන කොපමණ වාරයක් ඔබ සිතුවාද? (In the last week, how often did you think of problematic things?) | එහෙම වුණේ නෑ (Never) | සමහර දවස්වලට (Sometimes) | බොහෝ වෙලාවට (Often) | නිතරම වගේ (Very often) |
| 4. **පසුගිය සතියේ**, කොපමණ වාරයක් පාසල් යෑමට අකමැති බවක් ඔබට දැනුනා ද? (In the last week, how often did you feel like not wanting to go to school?) | එහෙම වුණේ නෑ (Never) | සමහර දවස්වලට (Sometimes) | බොහෝ වෙලාවට (Often) | නිතරම වගේ (Very often) |
| 5. **පසුගිය සතියේ**, පන්තියේ අනෙක් ළමයින් සමඟ ඔබ කොපමණ වාරයක් පහසුවෙන් අමනාප වූවා ද (තරහා ගත්තා ද)? (In the last week, how often did you easily become annoyed with other children in your class?) | එහෙම වුණේ නෑ (Never) | සමහර දවස්වලට (Sometimes) | බොහෝ වෙලාවට (Often) | නිතරම වගේ (Very often) |
| 6. **පසුගිය සතියේ**, යමක් වහාම සාර්ථක නොවූයේ නම්, කොපමණ වාරයක් කලබල වූවාද? (In the last week, how often did you become impatient if something didn’t work out right away?) | එහෙම වුණේ නෑ (Never) | සමහර දවස්වලට (Sometimes) | බොහෝ වෙලාවට (Often) | නිතරම වගේ (Very often) |
| 7. **පසුගිය සතියේ** සිදු වූ දේවල් ගැන කොපමණ වාරයක් ඔබ තනියෙන් සිතුවාද? (In the last week, how often did you think about that have happened?) | එහෙම වුණේ නෑ (Never) | සමහර දවස්වලට (Sometimes) | බොහෝ වෙලාවට (Often) | නිතරම වගේ (Very often) |
| 8. **පසුගිය සතියේ**, යම් වැඩක් වෙනකල් බලා සිටියදී ඔබ කොපමණ වාරයක් නොඉවසිලිමත් වූවාද? (In the last week, how often did you become impatient while waiting?) | එහෙම වුණේ නෑ (Never) | සමහර දවස්වලට (Sometimes) | බොහෝ වෙලාවට (Often) | නිතරම වගේ (Very often) |
| 9. **පසුගිය සතියේ**, ඔබ කොපමණ වාරයක් පහසුවෙන් කලබල වූවාද? (In the last week, how often did you easily become upset?) | එහෙම වුණේ නෑ (Never) | සමහර දවස්වලට (Sometimes) | බොහෝ වෙලාවට (Often) | නිතරම වගේ (Very often) |
| 10. **පසුගිය සතියේ**, ඔබ කොපමණ වාරයක් ඉක්මනින් දුක් වූවාද? (In the last week, how often did you easily become sad?) | එහෙම වුණේ නෑ (Never) | සමහර දවස්වලට (Sometimes) | බොහෝ වෙලාවට (Often) | නිතරම වගේ (Very often) |
| 11. **පසුගිය සතියේ** ඔබට කොපමණ වාරයක් බඩේ කැක්කුම ඇති වූවාද? (In the last week, how often did you have a stomachache?) | එහෙම වුණේ නෑ (Never) | සමහර දවස්වලට (Sometimes) | බොහෝ වෙලාවට (Often) | නිතරම වගේ (Very often) |
| 12. **පසුගිය සතියේ**, හේතුවක් සිතා ගැනීමට නොහැකිවම කොපමණ වාරයක් ඔබ වෙහෙසට පත් වූවාද? (In the last week, how often did you grow tired without knowing why?) | එහෙම වුණේ නෑ (Never) | සමහර දවස්වලට (Sometimes) | බොහෝ වෙලාවට (Often) | නිතරම වගේ (Very often) |
| 13. **පසුගිය සතියේ**, කොපමණ වාරයක් ඔබට හිසරදය ඇති වූවාද? (In the last week, how often did you have a headache?) | එහෙම වුණේ නෑ (Never) | සමහර දවස්වලට (Sometimes) | බොහෝ වෙලාවට (Often) | නිතරම වගේ (Very often) |
| 14. **පසුගිය සතියේ**, කොපමණ වාරයක් ඔබට නිදා ගැනීමට අපහසු වූවා ද? (In the last week, how often did you have trouble falling asleep?) | එහෙම වුණේ නෑ (Never) | සමහර දවස්වලට (Sometimes) | බොහෝ වෙලාවට (Often) | නිතරම වගේ (Very often) |
| 15. **පසුගිය සතියේ**, කොපමණ වාරයක්, ඔබේ හදවත වේගයෙන් ගැහෙන වන බවක් දැනුණාද? (In the last week, how often did you have the feeling that your heart was beating fast?) | එහෙම වුණේ නෑ (Never) | සමහර දවස්වලට (Sometimes) | බොහෝ වෙලාවට (Often) | නිතරම වගේ (Very often) |
| 16. **පසුගිය සතියේ**, කොපමණ වාරයක් ඔබේ අත් හදිසියේම උණුසුම්වන ගතියක් හා දහඩිය දමන ගතියක් දැනුණාද? (In the last week, how often did you have warm and sweaty hands all of a sudden?) | එහෙම වුණේ නෑ (Never) | සමහර දවස්වලට (Sometimes) | බොහෝ වෙලාවට (Often) | නිතරම වගේ (Very often) |
| 17. **පසුගිය සතියේ**, ඔබට කොපමණ වාරයක් හොඳට නිදාගැනීමට නොහැකි වූවා ද නැතහොත් රෑ මැද අවදි වීමට සිදු වූයේද? (In the last week, how often did you sleep badly or wake up in the middle of the night?) | එහෙම වුණේ නෑ (Never) | සමහර දවස්වලට (Sometimes) | බොහෝ වෙලාවට (Often) | නිතරම වගේ (Very often) |
| 18**. පසුගිය සතියේ**, ඔබට කොපමණ වාරයක් ආහාර ගැනීමේ අපහසුතාවක් නොතිබුණත්, කෑම ගැනීමට තරම් පිරියක්/ ආසාවක් නැති බව දැනුණාද? (In the last week, how often did you have no appetite for food, although you had hardly eaten anything?) | එහෙම වුණේ නෑ (Never) | සමහර දවස්වලට (Sometimes) | බොහෝ වෙලාවට (Often) | නිතරම වගේ (Very often) |
| 19. **පසුගිය සතියේ**, ඔබට කොපමණ වාරයක් ඔක්කාරය ‌හෝ වමනය කිරීමේ අවශ්‍යතාවක් දැනුණාද? (In the last week, how often did you feel nauseous or feel like you had to throw up?) | එහෙම වුණේ නෑ (Never) | සමහර දවස්වලට (Sometimes) | බොහෝ වෙලාවට (Often) | නිතරම වගේ (Very often) |
| 20. **පසුගිය සතියේ**, ඔබට කොපමණ වාරයක් කරකැවිල්ල දැනුණාද? (In the last week, how often did you feel dizzy?) | එහෙම වුණේ නෑ (Never) | සමහර දවස්වලට (Sometimes) | බොහෝ වෙලාවට (Often) | නිතරම වගේ (Very often) |

**
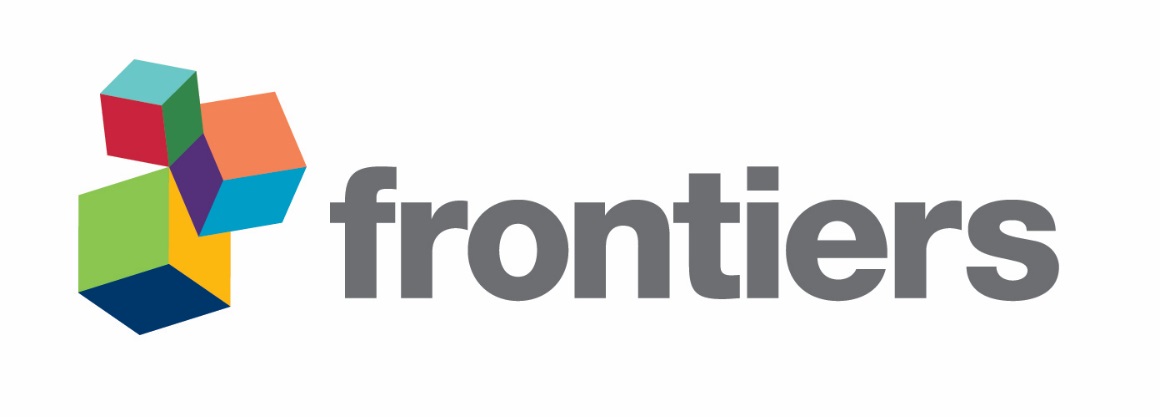
**

### Supplementary Figure 1.
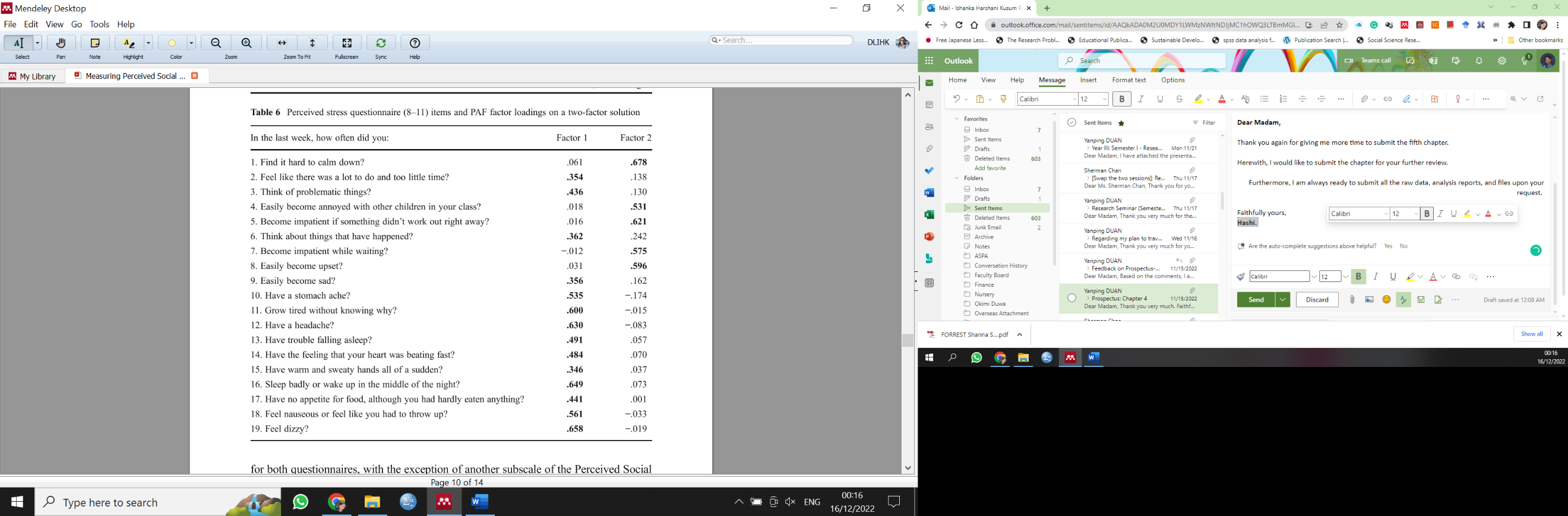
Factor loadings for PSQ8-11 original version

Source: Snoeren & Hoefnagels, 2014

.
